# Oncofertility and Fertility Preservation in Cancer Patients Across the Twitterverse

**DOI:** 10.3389/fendo.2022.926668

**Published:** 2022-06-29

**Authors:** Nayeli A. Martinez-Ibarra, Yuly A. Remolina-Bonilla, Hector H. Buerba-Vieregge, Regina Barragan-Carrillo, Francisco J. Castro-Alonso, Samantha Mateos-Corella, Maria T. Bourlon

**Affiliations:** ^1^ Department of Hematology and Oncology, Instituto Nacional de Ciencias Médicas y Nutrición “Salvador Zubirán”, Mexico City, Mexico; ^2^ Department of Internal Medicine, Hospital Regional de Alta Especialidad de Oaxaca, San Bartolo Coyotepec, Mexico

**Keywords:** oncofertility, fertility preservation, cancer, infertility, children, AYAs, Twitter ^®^

## Abstract

**Purpose:**

Infertility is a major problem affecting children, adolescents, and young adults (AYAs) with cancer, either due to the disease itself or because of oncologic treatment. Oncofertility (OF) focuses on counseling cancer patients about fertility risks and preservation options. However, OF and fertility preservation (FP) conversations on Twitter and their impact are unknown. We aim to characterize the users and type of content of these conversations.

**Materials and Methods:**

This observational study analyzed tweets with the hashtags “#Oncofertility” and “#FertilityPreservation” over eight months. We classified Twitter accounts by user type and country. Tweets were categorized by content type, and retweets and likes were quantified. Descriptive statistics were used for analysis.

**Results:**

A total of 399 tweets from 223 different accounts were evaluated. Twitter accounts comprised 22 countries and stemmed from high, upper-middle, and lower-middle-income countries in 86.5%, 5.4%, and 6.3%, respectively; no accounts from low-income countries were found. Accounts were mostly from physicians (37%) and healthcare centers (20%); we did not find any patient accounts. The most common content category was informative tweets directed to patients (30.8%), followed by discussion/sharing of medical papers (25.6%). Only 14.5% of tweets contained information about children and adolescents. Still, only 4.5% were aimed at children. Retweets were absent in 16.5% of the tweets, and 80.7% did not have comments.

**Conclusion:**

OF and FP discussions on Twitter were limited to interactions among medical professionals. Also, advocacy groups showed limited activity on social media. Even though a significant proportion of tweets directed to patients were found, no active involvement of patients was observed. Finally, limited number of tweets (4.5%) were directed to children and adolescents. There is a need to raise awareness about the effects of cancer on fertility in this group. Currently, Twitter is not a resource of information for children and AYAs with cancer who need OF counseling and fertility preservation. Our results open a debate on how to promote the use of social media in the future to improve the quality of OF information available, awareness, and care since there is an unmet need for fertility preservation access in young cancer patients.

## 1 Introduction

During the last decade, overall cancer incidence has increased among children, adolescents, and young adults (AYAs), but mortality has declined (1, 2). With successful treatments and increased survival, patients develop long-term treatment related toxicities. Radiation therapy and chemotherapy can destroy ovarian or testicular tissue and disrupt sex hormone production, increasing the risk of infertility (3). Patients have expressed limited knowledge and distress on fertility impact of cancer therapy (4–7). Patients and their families should receive an individualized assessment of gonadotoxic risk as early as possible after cancer diagnosis, and timely interventions should be performed to protect their reproductive goals. Oncofertility (OF) focuses on providing information and discussing the fertility issues, managing related complications, and bringing fertility preservation (FP) options to patients to maintain their reproductive potential (8). In recent times, OF has finally become a firmly established discipline and has been stated as a universal right (9).

Children and AYAs with cancer are the focus of OF counseling, and they report a higher need for information in virtual media (10). Currently, social media is increasingly uptaken by patients to obtain medical information and has become a burgeoning means of interaction between healthcare providers, healthcare centers, patients, and caregivers (11–15). Almost one-third of patients use social media for health-related reasons, including information, advice, and social support (11). In 2021, approximately 70% of American adults reported using any social media platform. YouTube and Facebook are the most employed, but there is an increase in the popularity of Twitter, especially among young adults. AYAs have the highest rates of social media use among any age group. Users aged between 18- and 29-years account for the 39% of Twitter users (16). This platform could empower young patients’ by increasing their medical knowledge and encouraging them to discuss their doubts and decisions with doctors. Informed patients have better disease awareness, higher adherence, and thus better clinical outcomes (17–19). Patients’ digital resources also increases patients’ participation in advocacy groups, helping others with the same condition and enhancing their satisfaction (20).

Cancer specific online communities follow particular interests and thus, interactions between diverse stakeholders including patients, families, healthcare providers, advocates, and policymakers take place. Several studies have examined the content of Twitter conversations regarding cancer, primarily discussions about specific tumors like breast, prostate, and lung cancer, which are the leading cancers among men and women globally (21–26). Cancer information on Twitter includes awareness, prevention/risk information, advice seeking, emotional support, cancer treatments, as well as disease outcomes and expectations (23–26). These interactions provide opportunities for non-clinicians, oncology professionals, cancer patients, and those who assist them to share information, advice, and support.

There are no published reports examining OF users and FP conversations in Twitter, nor users’ characteristics pertaining to discussions about OF. The aim of this study was to explore this field and its content on Twitter, determine the demographics of the origin of the tweets, the dissemination and impact generated by the shared information, and assess who is tweeting about it.

## 2 Materials and Tweets

Twitter is an information network made up of short messages known as “tweets” with a 280-character limit with over 206 million daily active users worldwide reported in 2021 and increasing daily (27, 28). Tweets can be liked, forwarded (“retweeted”), and replied. When users want to connect to other tweets containing a specific word or topic, they must look for a hashtag (a keyword or phrase preceded by the # symbol) (27). Cancer information on Twitter includes awareness, prevention/risk information, advice seeking, emotional support, cancer treatments, as well as disease outcomes and expectations (23–26).

In contrast with other malignancies with solidified social media outlets, such as breast (#BCSM), prostate (#PCSM) and lung cancer (#LCSM), Oncofertility has not a fully established presence in social media (15). We limited our search to evaluate the #Oncofertility and #FertilityPreservation hashtags as we considered those were the two that would be more accessible and trackable for the overall population. Indeed, including other hashtags in our search (as #Cancer), would yield a higher sensitivity, but will lower the specificity for the indented research question.

This was an observational study. Twitter’ search engine was used to find tweets. Original tweets and cited tweets with the hashtags of interest (#Oncofertility and/or #FertilityPreservation in cancer), in English, with any type of format (text, image, and video), posted within the period between January 1st to August 31st, 2020, were included. Tweets that did not meet the inclusion criteria or had incomplete information were excluded, and duplicated tweets were eliminated.

Accounts from each tweet were classified based on their profile information into the following categories: 1) physicians; 2) other healthcare providers (nurses, psychologists, and fertility counselors); 3) healthcare centers (hospitals, clinics, etc.); 4) professional organizations and societies; 5) medical journals; 6) continuing medical education; 7) patient education; 8) advocacy; 9) patients and 10) miscellaneous (accounts not able to be classified). The medical specialty was also considered. The author’s accounts’ country of origin was documented and classified based on the 2021 World Bank Data group classification (29). Each tweet’s type of content was categorized according to previously published studies (26, 30–32).

All tweets were independently reviewed and classified by two reviewers into one of the following categories: 1) discussion/sharing of papers published in medical journals 2) networking among healthcare professionals; 3) diffusion, sharing, and discussion of meeting presentations and/or invitations to webinars; 4) information for healthcare providers; 5) information for patients; 6) opinions/experiences tweeted by personal accounts; 7) others.

Inter-rater agreement was calculated with Cohen’s κ-coefficient. All hashtags contained in each tweet were captured and reviewed to evaluate which ones were the most related to our keywords. Public metrics for each tweet (number of retweets, likes, and comments) were collected to assess their dissemination potential. Descriptive statistics and Chi-square test were used for statistical analysis using SPSS version 25 (SPSS, Chicago, Illinois).

## 3 Results

### 3.1 Source of Tweets

**Figure 1:**
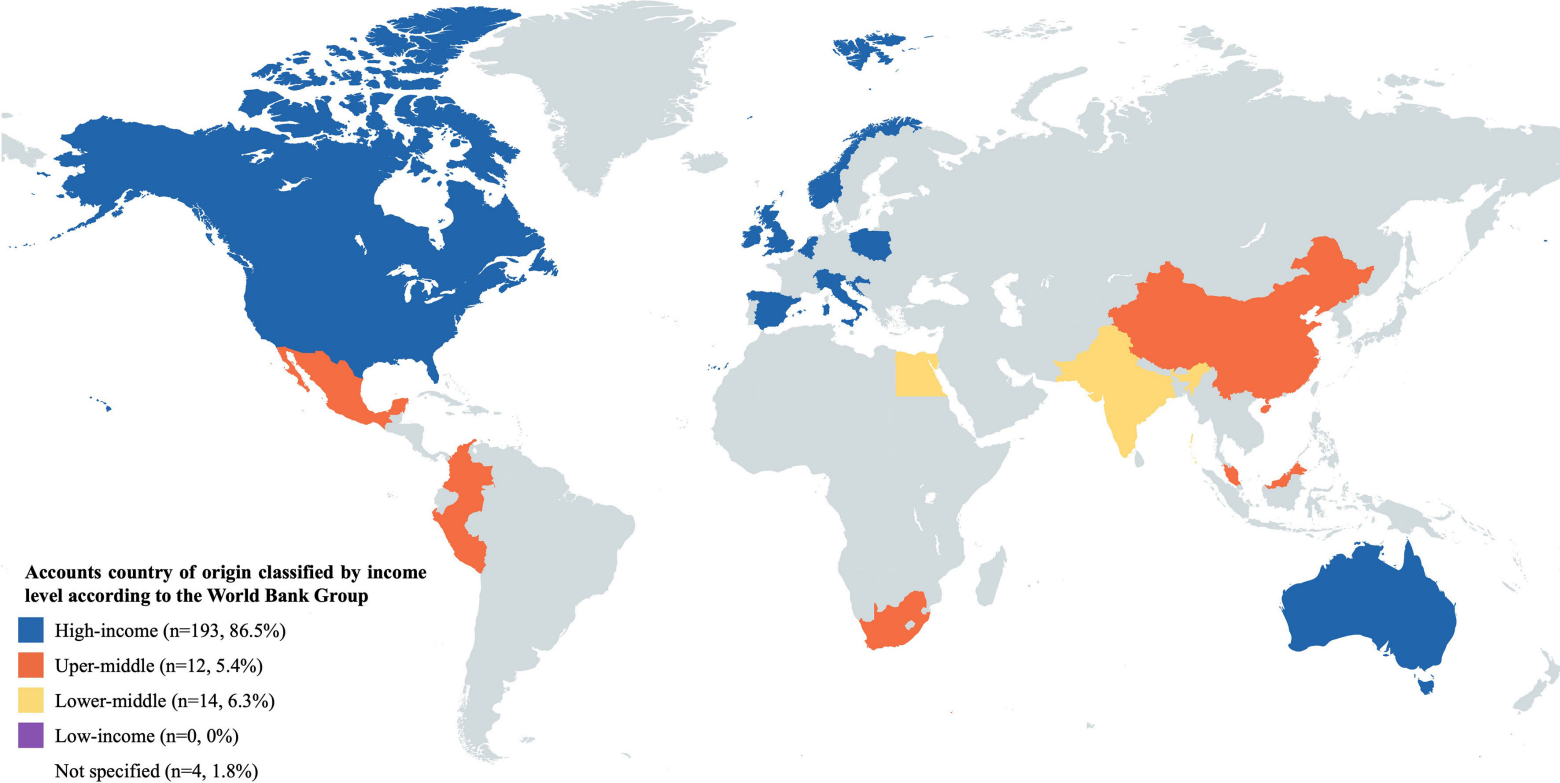
Country of origin of Twitter accounts of participants in OF and FP tweets classified by income level according to the World Bank Group.

A total of 674 tweets were initially captured. After the elimination of duplicates, 399 individual tweets were reviewed. All tweets were written in English and came from 223 accounts. We were able to classify accounts’ country of origin in 98.2% of cases. Accounts from 22 countries were documented. According to the World Bank group, classification came from high, upper-middle, and lower-middle-income countries, in 86.5%, 5.4%, and 6.3% respectively. No accounts from low-income countries were found **(** **Figure 1** **)**. Similar to the accounts’ country of origin, most tweets originated from high-income countries. A complete list of each country’s contribution can be found in **Supplement Table 1**.

**Figure 2:**
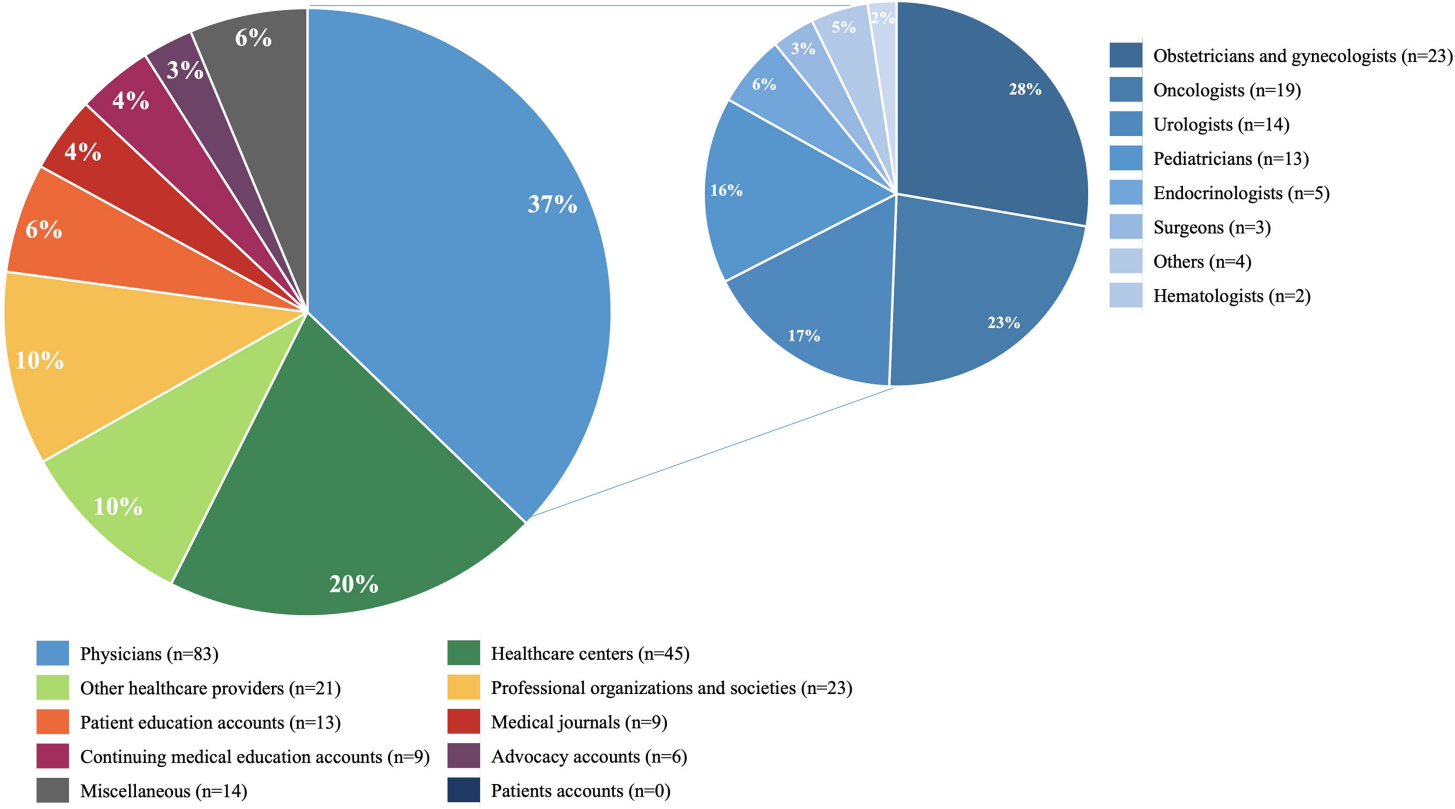
Distribution Twitter accounts according to the type of user holder (larger pie) and medical specialty distribution if the user was a physician (smaller pie).

The highest percentage of accounts belonged to physicians (37%), followed by healthcare centers (20%), other healthcare providers and professional organizations and societies (10% each one), patients education accounts (6%), continuing medical education accounts (4%), medical journals (4%) and advocacy accounts (3%); miscellaneous accounts comprised 6% of cases and no accounts from patients were found. Among the physician accounts’ subgroup, the most common identifiable medical specialties were Obstetrics & Gynecology (28%), followed by Medical Oncology (23%) and Urology (17%) **(** **Figure 2** **)**.

Gender could be determined in more than half of the accounts (51.5%), of those, 60.8% were women and 39.2% were men. Women tended to tweet more about personal opinions and experiences (70.5%), sharing/discussion of medical papers (35.3%) and information for healthcare providers (32.2%) than men (p= 0.036).

### 3.2 Tweets Content Analysis

Type of content was classified for all 399 tweets, with an inter-rater agreement of 83% (κ = 0.83; P = <0.001). Concordance was higher for tweets including discussion/sharing of papers published in medical journals (92.3%) and for the dissemination of information for patients (90.3%), and lowest for networking among healthcare providers (77.8%). **Table 1** shows the proportion of each tweet type of content and a representative example. Most tweets were about the dissemination of information for patients (30.8%), followed by the discussion/sharing of papers published in medical journals (25.6%), information for healthcare providers (14.8%), opinions/experiences tweeted by personal accounts (11.0%), sharing and discussion of meeting presentations and/or invitations to webinars (7.5%) and lastly, networking among healthcare providers (5.5%). There was insufficient information to classify 4.8% of tweets. About the tweets that were directed to patients (n=123, 30.8%), most of them contained general information (n=82). There were 22 tweets intended at women, one directed at men, and 18 directed at children and adolescents (**Table 2**).

**Table 1:** Classification of OF and FP related tweets according to their content and a representative tweet of each category.

Type of content	n=	%	Representative tweet
**Information for patients**	123	30.8	Some cancer treatments may affect #fertility, but there are preservation options. Before treatment begins, ask how it may affect your fertility, and discuss concerns with your health care team. https://fal.cn/385og#oncofertility #infertility #womenshealthweek
**Discussion of medical papers**	102	25.6	Great series of articles in @XXXXX on #fertility preservation in #cancer patients: #oncofertility is a universal right and a #GlobalOncology priority. Congrats to all the authors, very well done! @XXXXX @XXXXX #OncoAlert @XXXXX https://ascopubs.org/doi/full/10.1200/GO.19.00337#.Xl_yE6437Eg.twitter
**Information for healthcare providers**	59	14.8	Comparing Options for Ovarian Tissue Cryopreservation to Preserve Fertility in Pediatric Patients With Cancer https://ascopost.com/news/january-2020/ovarian-tissue-cryopreservation-to-preserve-fertility-in-pediatric-patients-with-cancer/#pedonc #oncology #cancer #oncofertility
**Personal opinions/experiences**	44	11.0	RT “Preserving fertility in cancer during a crisis may sound “elective”, but to the young adult with cancer, it can mean hope in the face of a future clouded by uncertainties. Let’s safely care for this vulnerable population while upholding our social obligation. @XXXXXX” I wholeheartedly agree! #oncofertility #ChildhoodCancer #AYAcancer
**Meetings’ diffusion**	30	7.5	The next #fertilityfocus takeover will be on the 16th of July with the brilliant @XXXXX, Join XXXXX as he takes over the Urology News handle to discuss oncofertility. If it’s like his last takeover, it’s going to be good. #Fertilitythursday
**Networking among healthcare professionals**	22	5.5	Are you a service provider working with people with cancer? Can you spare 10 minutes to take our survey? #oncology #alliedhealth #ruralhealth #psyonc #supponc #oncologynurses #oncofertility #lgbtonc #oncorn #radonc #surgonc #radonc #onconav
**Others**	19	4.8	POWER THROUGH: cancer at age 3; e-learning at age 10…what kind of mom do you think she will be? #ChildrenSurvivingCancer #Oncofertility

“XXXXX” was used to censor users’ account to protect their privacy.

**Table 2:** Tweets with information directed to patients and examples of tweets directed to, women, men and children and adolescents.

	n=	%	Representative tweet
**General information**	82	66.7	Some cancer treatments may affect #fertility. Before treatment begins, ask how it may affect your fertility and discuss concerns with your health care team. http://bit.ly/FertilityConcern #oncofertility #infertility #womenshealth #IWD2020
**Women**	22	17.9	With the field of medicine advancing every day, #oncofertility joins the two fields of oncology and #gynecology to provide cancer survivors with the chance of increasing their reproductive level. To know more visit- https://buff.ly/2NUnbej
**Men**	1	0.8	If you have a cancer diagnosis, did you know you may be able to freeze your sperm before treatment? Let’s start increasing awareness of the #fertilitypreservation option #WorldCancerDay
**Children and adolescents**	18	14.6	Did you know treatment for pediatric cancers is a common cause of infertility? Help me promote #oncofertility awareness and support for this amazing event. Only a few tickets left #sharethelove @XXXXXXX https://instagram.com/p/B70qb_8lP4I/?igshid=3iqov27e33ee

Links to websites were included in 66.6% of the tweets and 69.4% contained at least one image or video. Statistical differences regarding the type of content and tweet authors were found (p<0.001) as well as in medical specialties (p=0.006). In the category of discussion of papers, 50% of tweets were posted by physicians and 17.6% by medical journal accounts. Networking tweets were commonly created by professional organizations and societies (68.2%), information for patients tweets by institutes or medical centers (41.5%), and diffusion of meeting or webinars, information for healthcare providers and personal opinions tweets mainly by physicians in 30.0%, 35.6% and 65.9%, respectively.

### 3.3 Reach and Dissemination

The median number of all tweets per user was 9.8 (range 11 – 225,700), and the median number of OF and fertility preservation-related tweets per user was 2.

The median users’ followers were 13,594 (range 9 – 1,267,484), whilst the median of followed accounts was 1,041 (range 0 – 14,600). We found 19 accounts with less than 100 followers (8.5% of users) and 24 accounts with less than 100 followed accounts (10.7%). Only 2 accounts had 0 followers (0.8%). Therefore, the probability of fake accounts is considered low (≤1%).

Among OF tweets, the median number of retweets was 2 (range 0 – 95), with a total of 802 retweets, whereas the median number of likes was 6 (range 0 – 66), with a total of 2,488 likes.

A great percentage of the tweets (47.3%, n=189) did not receive any retweet; 16.5% (n=66) received one retweet, 10.5% (n=42) received two retweets and 15.0% (n=60) received three or more retweets. We found that 23% (n=92) of tweets did not receive any likes, 25.0% (n=100) received at least one or two likes and 41.8% (n=167) received three or more likes. Concerning comments or replies, most of the tweets (80.7%) did not get any, 11.5% received one reply, and 7.8% received at least two or more replies.

**Figure 3:**
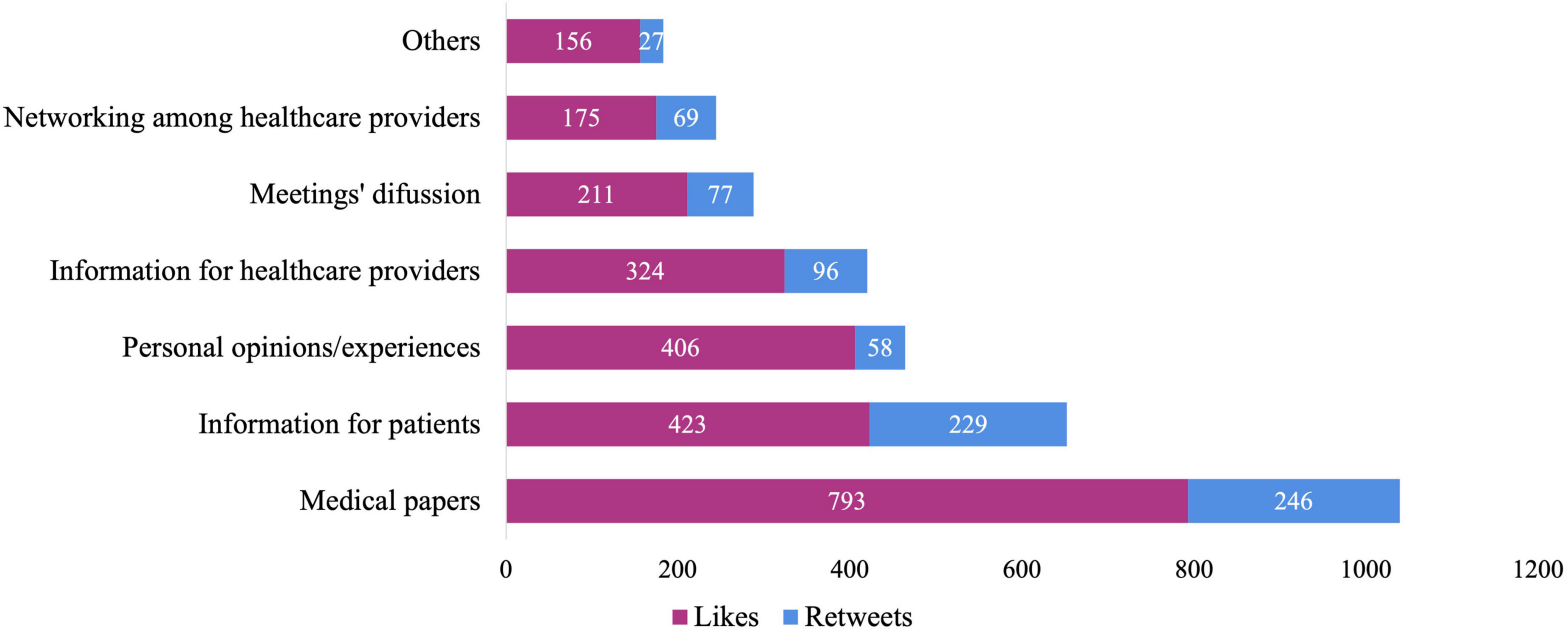
Reach and dissemination of tweets organized by content.

Among 802 retweets, the majority (n=246) belonged to the discussion/sharing of papers published in the medical journals category; the least retweeted category was personal opinions/experiences (n=58). As to likes, at the top of all the categories, we found the discussion/sharing of papers (n=793), followed by dissemination of information to patients (n=423), and the least liked category was networking among healthcare professionals (n=175) **(** **Figure 3** **).** The most frequently liked and retweeted tweets were posted by physicians (34.6% and 34.6%, respectively), followed by professional organizations and societies (20.1% and 20.1% respectively). We found no statistical differences in content, authors, and dissemination surrogates (retweets, likes, commentaries) in the participants’ countries according to World Bank income level since most of them were high-income countries.

The hashtags more associated with our keywords were #Cancer, #Fertility, #Infertility, and #FertilityMatters. The hashtag with the COVID-19 word was included in 5.2% (n=21) of tweets.

### 3.4 OF and FP Information Shared on Twitter Regarding Children and Adolescents

**Figure 4:**
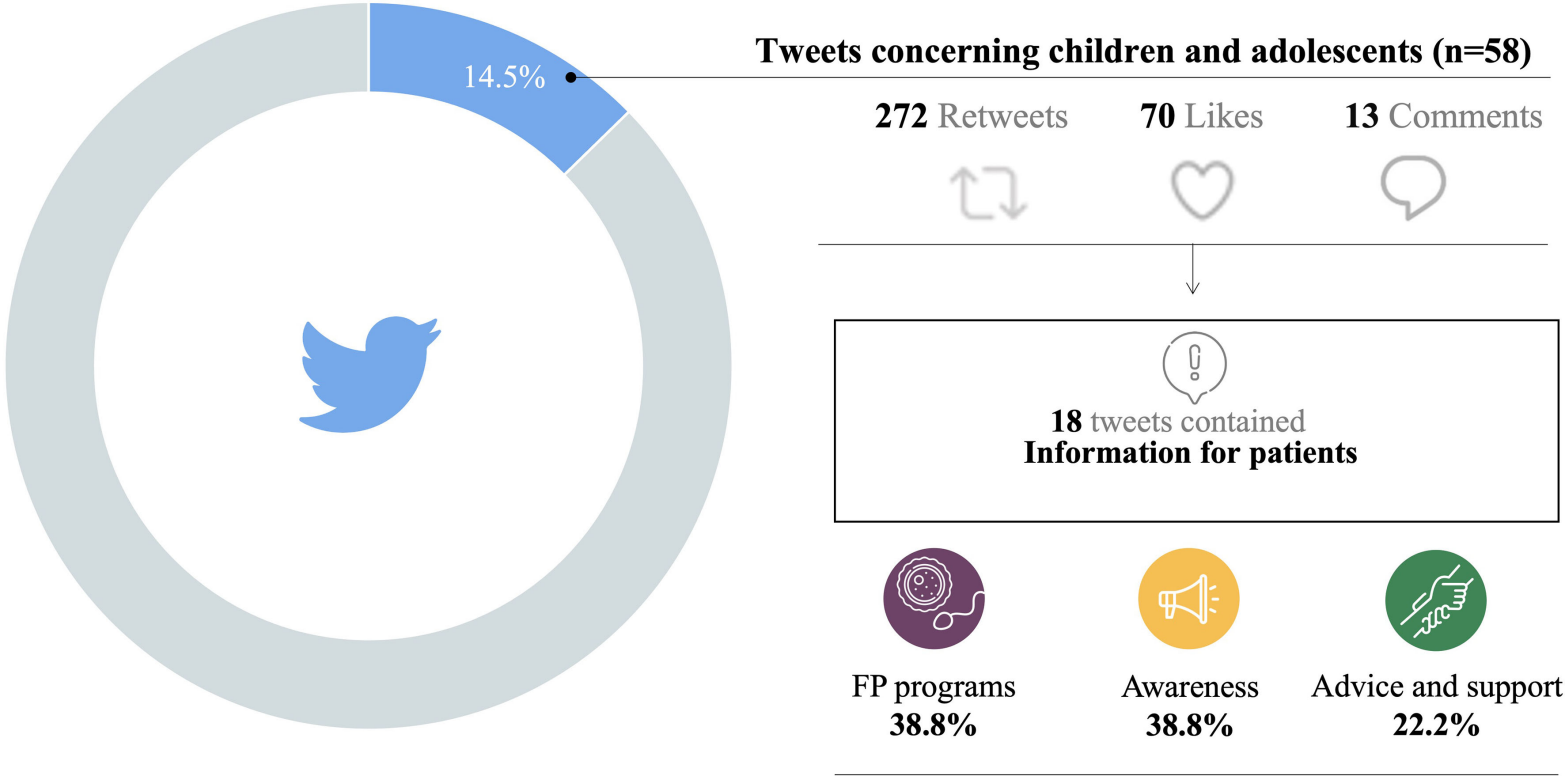
OF and FP information shared on Twitter regarding children and adolescents.

A total of 58 tweets (14.5%) were assessed from 47 distinct accounts from 8 different countries. The most common author category (n=19, 40.4%) was physicians and the predominant country of origin of the accounts was the United States (n=19, 72.3%). The most popular type of information shared was discussion/sharing of papers published in medical journals (n=18, 31%) and dissemination of information for patients (n=18, 31%), tweets belonged to this category were categorized into awareness (n=7), FP programs (n=7) and advice and support (n=4) **(** **Figure 4** **)**.

These tweets received 272 likes and 70 retweets, with 49 of them (84.5%) receiving no response. The discussion/sharing of papers published in medical journals received 99 likes and 38 retweets, while the dissemination of information for patients received 81 likes and 23 retweets.

## 4 Discussion

To our knowledge, this is the first study exploring OF and FP tweets, and impact generated. Most tweets belonged to accounts from high-income countries, which aligns with the adoption, widespread, and dissemination of OF in these countries. Social media use is beyond national wealth and internet availability and is significantly linked to the population’s age: a youthful population (i.e., the AYA-age group) contributes to greater social media use in developing countries *vis-à-vis* countries with higher incomes but with a low rate of social media adoption and a more aged population (33).

Low-income countries’ lack of engagement may be explained by more than just their income level. The disparities in the income level participants’ countries reflect known barriers in the equitable access to the OF field, barriers beyond internet access. Such obstacles include a lack of referral pathways, cost-based access limitation, limited health literacy, lack of training or awareness among medical professionals, cultural or religious constraints, and a lack of consensus about the best way to deliver information to patients (34, 35). Recent clinical practice guidelines published by the PanCareLIFE Consortium state interventions to overcome the barriers, among which written, and online educational resources have shown to increase discussion rate of infertility risks and options to preserve fertility to AYA cancer patients (36). We observed an important participation of India in our study **(** **Supplement Table 1** **),** it was the fourth country to tweet about OF and FP. In the field of OF, India has shown a significant interest in overcoming several barriers to improve scientific knowledge, service delivery, advocacy, and research efforts (37).

Almost 25% of patients aged 10 to 12 years old and nearly 40% of patients aged 16 to 18 years old expressed a greater need for cancer education (38). We need to increase cancer knowledge and reduce the fear and stress caused by these patients’ future and we believe that Twitter with its large proportion of young users is a powerful social media tool for starting conversations about fertility concerns; thus, the OF community should make direct efforts to leverage discussions in this setting. Social media can help to bridge socioeconomic disparities.

OF conversations were dominated by physicians and mainly were about medical papers. The use of social media by doctors and healthcare providers is well established: surveys report that 72% of oncology physicians or trainees use these platforms for professional development and networking (39). In our study, oncologists were the second most common specialty among physicians’ accounts. The popularity of Twitter among oncologists is constantly growing. According to a survey of Canadian oncology physicians and trainees, 72% of respondents used social media (40). Oncologists’ involvement in this social media platform has been established in studies published in the Journal of Oncology Practice, with oncologists updating, educating, and expanding knowledge transmission of credible evidence-based information (39, 41). Oncologists on Twitter also promote active patient involvement in cancer discussions (42). Oncologists are expected to participate in OF tweets because they are the ones who prescribe cancer treatments and oversee the implications of treatment side effects during the follow-up and are more conscious about these issues compared with other medical specialists.

The dissemination of information directed to patients was the most popular category, nevertheless we found no active participation of patients in these conversations, nor tweets authored by patients in the study period. The lack of involvement of patients in our study shows a significant contrast with previously published analyses about Twitter use in other oncology fields. For example, Twitter discussions dealing with breast, lung, prostate, and kidney cancer show a highly engaged community in which most tweets are authored by patients, cancer survivors, and family members. In addition, content is mainly related to cancer diagnosis, treatments, and their side effects and tweets looking for guidance and support (12, 22–26).

The lack of engagement regarding OF social media with the AYA was not expected, as prior reports mentioned that most social media users are aged between 18 and 29 years. Nonetheless a lack of uptake and acceptability of health promotion on social media has been low among young people, with an average of engaged AYA participants ranging from 5 to 15% (43). We hypothesize that AYA’s social media use is more of an outlet from their disease than a tool for health-related information access or sharing. Social media use for health-related reasons in AYAs remains a controversial topic. Even though a great amount AYAs use social media, only a small percentage (3.5%) use it for seeking health information. The health-information they tend to look for in social media is about fitness and sexual health (44). Most of the time they spend on their cellphone is because they are passing time or connecting with friends and family (44, 45).

Current social media platforms availability must be also considered. For its nature dependent on words rather than interactive media, Twitter is less attractive to young patients and the general population as a source for learning (23). Young adults found interactive media (for example, video format) easier to learn from. They stated that they could identify accurate YouTube health content and felt that the presenter was honest and relatable. The former contrasts with Twitter, which was rarely used as a source for health information (46).

In addition, only 4.5% (n=18) of all tweets were directed to this population bringing information about FP programs, awareness, and advice and support stressing the need for information created to inform patients, rather than exclusively scientific content aimed at physicians.

As for children with cancer, direct social media interaction is highly unlikely though Twitter, so efforts are aimed at their parents about informing potential infertility risk and OF assessment. Even though we found some tweets aimed at informing parents of children with cancer about infertility risk a similar lack of interaction was observed, like AYAs. In this population, Facebook was the most widely used social media platform by a wide margin; 78% of parents reported using it every day, but only 2% using Twitter daily (47).

We found little engagement of advocacy accounts (3%). OF advocacy groups should take advantage of this platform to improve their reach among potential patients and family members.

Greater participation of females compared to male users was observed. This phenomenon has several potential explanations. The estimated number of new cancer cases in AYA women almost doubled the cases in AYA men in 2020 (8.7% and 4.4%, respectively), thus having numerically more fertility concerns (21). Moreover, FP strategies for females are more technically demanding and time-intensive than those required for male patients, with higher complication rates and lower chance of success which varies between 40 to 61.9%. FP in females also depends on age at retrieval, number of oocytes, and technique (48–51). The complications rates of oocyte retrieval are less than 0.5%, however, these complications can be severe, and life-threatening and should not be underestimated (52). These difficulties might drive female treating physicians to be more likely to discuss fertility issues with their patients, as some studies have shown (53, 54).

Our study has several limitations. There are no standardized methods to perform an analysis of social media; in specific tumor types like breast and prostate cancer, social media traffic is substantially different among different time frames (55). Prior medical research using Twitter have utilized observation timeframes from 22 days to 12 months (32, 56). We considered that the collected information during our timeframe provides a realistic scope of current social media use. As this is the first study of its nature, we considered that these results are vital for the OF community and key for designing interventions to improve social media engagement in AYAs.

Furthermore, Twitter’s search engine may have some limitations in the access of all the tweets searched with the hashtag of interest, like a misspelling. Using English keywords might ignore information in other languages, so equivalent words in other common tongues should be performed. Other social media platforms could be used as a dominant way of information, but the way they are designed to make them less trackable than Twitter. Finally, OF and FP conversations may start on Twitter and then migrate to verbal in-office discussions, which cannot be followed by this study.

Share more appealing information for patients and caregivers, such as infographics and videos, promote their active participation in Twitter using surveys to find out what their interests are, and create hashtags and communities to facilitate patient access to specific information are some strategies that could help us in increase knowledge, education, and engagement of patients and caregivers.

Future directions are needed to explore and understand how social media can be used and exploited by physicians, other healthcare providers, advocacy groups, patients, and their families for exchanging information. There is ample opportunity among the OF community (especially to patients and advocates) to use Twitter as a means to improve their reach and leverage fertility discussions.

## 5 Conclusion

Oncofertility and fertility preservation discussions in Twitter were limited to interactions among medical professionals and medical centers, while limited participation of advocacy groups and no active involvement among patients were observed. From a Global Oncology perspective, most tweets came from high-income countries, with limited participation of middle-income countries and a total lack of participation in low-income countries.

The Oncofertility community needs to implement initiatives directed to create more appealing social media content that captures patients’ attention, facilitates their engagement in decision-making, and improves their long term well-being in survivorship. There is a need to raise awareness about the fertility impact of cancer in children and AYAs. These results open the debate whether social media could be used in the future to improve the quality of oncofertility care and suggest that exists a need to identify strategies to increase fertility education for cancer patients and their families.

## Data Availability Statement

The raw data supporting the conclusions of this article will be made available by the authors, without undue reservation.

## Ethics Statement

All procedures performed in this study were in accordance with the ethical standards of the institution. There were no human subjects as part of this manuscript and, therefore no need for informed consent.

## Author Contributions

Conception and design: MB; Data collection: NM-I, SM-C, and HB-V; Analysis and interpretation of data: NM-I, YR-B, HB-V, RB-C, FC-A, SM-C, and MB; Manuscript writing and approval of final article: All authors contributed to the writing of the manuscript. All authors read and approved the final manuscript.

## Conflict of Interest

The authors declare that the research was conducted in the absence of any commercial or financial relationships that could be construed as a potential conflict of interest.

## Publisher’s Note

All claims expressed in this article are solely those of the authors and do not necessarily represent those of their affiliated organizations, or those of the publisher, the editors and the reviewers. Any product that may be evaluated in this article, or claim that may be made by its manufacturer, is not guaranteed or endorsed by the publisher.
