# Supplementary material for: Oncofertility and Fertility Preservation in Cancer Patients Across the Twitterverse
Source: Front Endocrinol (Lausanne). 2022 Jun 29;13:926668. doi: 10.3389/fendo.2022.926668 (PMC9278620; doi:10.3389/fendo.2022.926668)
Supplement: Supplementary file 1 [file DataSheet_1.docx]

***Supplementary Material***

**Supplementary Table 1** Classification of tweets by income level group according to the World Bank

| Income level classification | Tweets generated  (n=399) | % |
| --- | --- | --- |
| High-income countries^1^ | 354 | 88.7 |
| Upper-middle-income countries^2^ | 16 | 4.0 |
| Lower-middle-income countries^3^ | 18 | 4.5 |
| Low-income countries | 0 | 0 |
| Not specified | 11 | 2.7 |

**_1_**_.United States (n=259), United Kingdom (n=33), Australia (n=18), Canada (n=11), Italy (n=10), Spain (n=6), Ireland (n=4), Poland (n=3), Netherlands (n=3), Belgium (n=2), Norway (n=2), Switzerland (n=2), Croatia (n=1)_

**_2._** _Mexico (n=5), Malaysia (n=4), South Africa (n=3), China (n=2), Colombia (n=1), Peru (n=1)_

**_3._** _India (n=15), Pakistan (n=2), Egypt (n=1)_

***Appendix***

**Glossary section**

- **Hashtags:** A hashtag is a type of tag used to describe topics on social networking websites, most notably Twitter. Hashtags, like all tags, are a type of metadata (data about data). Twitter has popularized the use of the term hashtag. Hashtags give other users and indication what a particular tweet is about. Hashtags are indicated as such because they are prefixed with the pound symbol (#).
- **Reply:** The Twitter @reply method is used when replying to a user via the format: @username message, where "username" is the recipient's Twitter handle. Another way to reply to a message is simply by clicking the tweet reply button. Message replies are received and saved under the Twitter @Connect tab. The @reply feature is also known as a mention.
- **Retweet (RT):** refers to the act of sharing or reposting other users' tweets and posts on the social networking website called Twitter. In order to retweet a tweet, the user has to click the Retweet link, which will automatically repost that existing tweet on the user's feed and provide attribution to the original poster.
- **Social Media:** is a catch-all term for a variety of internet applications that allow users to create content and interact with others.
- **Tweet:** is a Twitter message displayed on a user's profile page, which is publicly visible by default, and shared with all his or her "followers." It can be described as a status update or post published by a Twitter user. Tweets are limited to 280 characters, including spaces, and may include URLs and hashtags.
- **Twitter:** is a social networking and microblogging online service that allows users to send and receive text-based messages called "tweets."
- **Twitterverse:** is social media jargon used to describe the collective number of members of online social media network Twitter. The Twitterverse refers to all Twitter users, regardless of their gender, location, and overall activity/tweets on Twitter.

**References**

Techopedia Dictionary. https://www.techopedia.com/dictionary [Accessed May 24, 2022].
